# Setting expectations following endoscopic cubital tunnel release

**DOI:** 10.1007/s11552-014-9629-7

**Published:** 2014-04-01

**Authors:** Tyson K. Cobb, Anna L. Walden, Peter T. Merrell, Jon H. Lemke

**Affiliations:** 1 Orthopaedic Specialists, Inc, 3385 Dexter Court, Davenport, IA 52807 USA; 2 Department of Research, Orthopaedic Specialists, Inc, Davenport, IA USA; 3 Genesis Medical Center, 1227 E. Rusholme Street, Davenport, IA 52803 USA

**Keywords:** Endoscopic cubital release, Anterior transposition of ulnar nerve, Comparison of outcomes, Recovery characteristics, Return to work, Cost analysis

## Abstract

**Background:**

The objective was to evaluate recovery characteristics of patients undergoing endoscopic cubital tunnel release (ECuTR) by determining the following: (1) return to work (RTW) times following ECuTR compared with RTW times of patients that underwent anterior transposition of the ulnar nerve (ATUN), (2) satisfaction rates and factors affecting satisfaction, (3) resolution rates of common preoperative complaints and findings, and (4) effect of preoperative ulnar nerve subluxation on postoperative outcomes.

**Methods:**

A total of 172 cases in 148 patients undergoing ECuTR were prospectively enrolled including 56 women and 92 men. Kaplan-Meier analyses were performed to determine RTW time for ECuTR patients and for a cohort of 15 patients that underwent ATUN. Patients were evaluated for subjective and objective complaints preoperatively and postoperatively. Cases were grouped by Dellon’s classification preoperatively and modified by Bishop’s postoperatively.

**Results:**

Half of ECuTR patients returned to normal work within 8 days postoperatively versus 71 days following ATUN. Variables significantly negatively affecting RTW were male sex, manual labor, and worker’s compensation status. Dellon’s was the best predictor of postoperative satisfaction. Complete resolution of symptoms occurred in 86 % of patients for weakness, 81 % for pain, 79 % for numbness and tingling (N/T), 78 % for atrophy, 76 % for abnormal two-point discrimination, and 65 % for Wartenberg’s. Preoperative ulnar nerve subluxation had no effect on outcome.

**Conclusions:**

Improved RTW time following ECuTR versus ATUN indicates potential and substantial cost-saving implications with respect to reduced worker productivity loss. Patients with more severe preoperative Dellon’s classification can expect less optimal results regarding postoperative satisfaction and resolution rates of N/T and pain.

## Introduction

Cubital tunnel syndrome (CuTS) is a compressive neuropathy of the ulnar nerve at the elbow. It has an estimated incidence of 18 to 25 per 100,000 person-years [1, 2]. A recent publication found that the number of ulnar nerve surgeries increased by 47 % over an 11-year time span [3]. Anterior transposition of the ulnar nerve (ATUN) was once the accepted gold standard surgical procedure for idiopathic CuTS. More recently, however, simple decompression has steadily gained support [4–15]. According to the findings of a Cochrane review of multiple level 1 trials comparing simple nerve decompression with ATUN, there was no significant difference in efficacy between the two procedures and a lower complication rate following simple decompression [6]. Simple decompression has also been shown to be more cost effective than ATUN [16]. The equivocal differences in outcomes, higher-complication rates for ATUN, and cost effectiveness analyses suggest that simple decompression is a favorable surgical procedure for idiopathic CuTS [4–6, 17].

Endoscopic cubital tunnel release (ECuTR), the newest of the surgical options for simple ulnar nerve decompression at the elbow, has been described by several authors using a variety of techniques [18–32]. This minimally invasive procedure utilizes an endoscope for visualization through a small incision. It entails a reduced soft-tissue dissection compared with traditional approaches and thereby potentially allows for a more rapid recovery with minimal scarring. A study comparing ECuTR with open in situ decompression of the ulnar nerve found significantly higher complication rates and reduced satisfaction following open decompression compared with the endoscopic group [24]. Although ECuTR is minimally invasive and thus should allow faster return to work time, little evidence is available in the current surgical literature to support this hypothesis. Likewise, there is little to guide clinicians on how to advise patients regarding expected outcomes of satisfaction and resolution rates for common preoperative findings and symptoms. Ulnar nerve instability is frequently considered a contraindication for simple decompression because of the perceived risk of painful instability after decompression that could necessitate revision surgery with ATUN. However, there is currently little supporting evidence in the literature. Furthermore, it should be noted that ATUN has a higher incidence of postoperative complications [6, 24, 33, 34]. The purpose of this study was to evaluate and provide evidence for recovery expectations following ECuTR by determining the following: (1) return to work (RTW) time following ECuTR with a retrospective comparison with a cohort of patients that underwent ATUN, (2) satisfaction rates and factors affecting satisfaction, (3) resolution rates of common preoperative complaints and findings, and (4) the effect of preoperative ulnar nerve subluxation on outcomes.

## Materials and Methods

Necessary and appropriate consent was obtained from each patient, and the study protocol conformed to the ethical guidelines of the 1975 Declaration of Helsinki as reflected in prior approval by a local institutional review board for this study, a case series of patients undergoing ECuTR with a retrospective comparison for RTW times of patients who underwent ATUN. Although this study design incorporates a retrospective comparison group, all ECuTR data were collected prospectively. Our institution, a private orthopedic group practice, established and utilized an ECuTR registry whereby all consenting patients scheduled for ECuTR were prospectively enrolled.

Diagnosis of CuTS was determined by the treating surgeon based on patient-reported history of paresthesia or numbness in the ulnar nerve distribution, positive physical findings including Tinel’s sign over the ulnar nerve at the elbow and elbow flexion-compression test as well as positive nerve conduction studies, which were conducted and analyzed by electrophysiologists.

In the absence of clinical progression, patients continued conservative management including nighttime splinting, avoidance of provocative activities, nerve glide exercises [35], and nonsteroidal anti-inflammatory drugs as long as tolerated. We offered patient surgery for idiopathic CuTS if they failed conservative treatment. Patients who presented with progressive clinical findings of atrophy, elevated two-point discrimination (≥6 mm), or static changes in the ulnar nerve distribution were offered surgery regardless of the length of conservative treatment.

Contraindications included the presence of a local mass or space-occupying lesion compressing the nerve, severe long-standing elbow contractures requiring release, subluxating ulnar nerves with prominent ulnar neuritis, and conditions necessitating ATUN such as humeral malunions with cubitus valgus or prior surgery or trauma with a scarred and adherent nerve. Subluxating nerve in the absence of ulnar neuritis was not a contraindication to ECuTR. Prominent ulnar neuritis was defined as severe ulnar nerve pain associated with ulnar nerve subluxation during elbow flexion. These patients frequently have a history of trauma, and pain symptoms are more prominent than numbness and tingling. Data collected relative to work type were categorized into three groups: (1) homemaker/retired/unemployed, (2) manual labor, and (3) nonmanual labor. RTW time was recorded for the date patients reported returning to normal activity following surgery.

Subjective and objective patient assessments were performed preoperatively and postoperatively at time intervals of 1 week, 1 month, 3 months, 6 months, 12 months, and yearly thereafter. An occupational hand therapist collected objective data.

A hand therapist, registered nurse, physician assitant, or physician recorded patient pain and satisfaction at each postoperative visit. Pain was rated on a scale of 0 to 10 (0 = no pain, 10 = worst pain imaginable). Satisfaction with outcome of the surgical procedure was rated on a scale of 1 to 5 (1 = very dissatisfied, 5 = very satisfied). Modified Bishop’s classification was calculated postoperatively [36].

During physical examination, ulnar nerve instability was considered positive when the ulnar nerve subluxed over the medial epicondyle during elbow flexion or relocated during elbow extension. Care was taken to attempt to rule out other structures potentially masquerading as a subluxing ulnar nerve such as triceps muscle or subcutaneous tissue [37]. Eliciting a positive Tinel’s sign over the subluxed structure confirms the subluxed structure to be the ulnar nerve.

A cohort of 15 patients undergoing ATUN for CuTS was reviewed retrospectively to compare RTW time with that of ECuTR. The ATUN cohort was selected by the same surgeon using the same criteria as the ECuTR cohort. To reduce the potential for selection bias, only patients who received ATUN prior to implementing ECuTR in the surgeon’s practice were included in the retrospective comparison group. ATUN was utilized for all CuTS surgical patients in our practice prior to implementing ECuTR. We believe this to be a reasonably fair comparison in that the two groups were selected based solely on calendar date of surgery and on a change in the surgeon’s practice and therefore represents typical CuTS patients presenting for surgery in private practice and treated by the same surgeon. Patients who received ATUN performed after implementation of ECuTR were not included for comparison because these patients were made-up of exclusions from the ECuTR procedure. None of the ATUN patients had ulnar neuritis. Practice policy and procedures enforce that RTW slips be written on carbon copy prescription pads and retained in the charts. RTW time was determined by reviewing the charts for RTW slips thereby reducing the chance of collection bias between the two cohorts.

ECuTR surgical technique was performed under general, regional, and local anesthesia with sedation or wide-awake anesthesia [38]. A standard 30-degree, 4-mm endoscope and the EndoRelease ECuTR system were used (Integra Life Sciences, Plainsboro, NJ). This system includes a cannula specifically designed for cubital tunnel release. The cannula has a flat undersurface that helps hold the ulnar nerve under the cannula. Slots on the inferior surface allow visualization and protection of the ulnar nerve during release. The cannula has an attached retractor which atraumatically holds the superficial nerves in a protected position during the release. Spatulas are available to facilitate placement of instrumentation into the canal. A complete description of the surgical technique has been published elsewhere [30]. The procedure used for the ATUN cohort was performed as previously described [11]. Postoperatively, both ATUN and ECuTR patients were encouraged to begin immediate ROM exercises. Neither of the groups were splinted. Both groups were encouraged to return to light duty the day after surgery and full duty as soon as tolerated. Patients in both groups were given additional time off if required.

### Statistical Methods

Sex differences of baseline symptoms were compared using Fisher’s exact tests (Table 1). Ordinal data (Dellon’s and Bishop’s ratings) [7, 39] were compared using exact permutation tests for ordered multinomial distributions. Kaplan-Meier survival analysis was utilized to determine the time to RTW postoperatively. Patients who underwent bilateral surgery who did not go back to work prior to the second surgery were censored at the date of the second surgery. Logistic regression models were used to predict resolution of pain and numbness and tingling (N/T). Ordinal logistic regression models were fit to predict cumulative probabilities for patient satisfaction, and then, the estimated probabilities were converted to mean scores.

**Table 1:** Comparison of preoperative status by sex (sorted by overall prevalence rate)

Preoperative condition	Women (*n* = 56)	Men (*n* = 92)	Exact test	Overall
	64 cases	108 cases	*P* value	172 cases
Dellon’s class—*n* (%)				
1—mild	6 (9.4)	14 (13.0)		20 (11.6)
2—moderate	30 (46.9)	40 (37.0)	*0.660*	70 (40.7)
3—severe	28 (43.8)	54 (50.0)		82 (47.7)
Subjective complaints (ulnar nerve distribution)—*n* (%)				
Numbness/tingling	63 (98.4)	98 (90.7)	*0.055*	161 (93.6)
Pain	45 (71.4)	49 (45.4)	*0.001*	94 (55.0)
Orthopedic exam (ulnar nerve distribution): positive findings—*n* (%)				
Tinel’s sign	57 (89.1)	97 (89.8)	*1.000*	154 (89.5)
Elbow flexion compression	60 (93.8)	82 (75.9)	*0.001*	142 (82.6)
Static numbness	23 (35.9)	60 (55.6)	*0.018*	83 (48.3)
Finger abduction weakness	31 (48.4)	44 (40.7)	*0.344*	75 (43.6)
Two-point discrimination ≥6 mm	18 (28.1)	31 (28.7)	*1.000*	49 (28.5)
Hypothenar muscle atrophy	13 (20.3)	34 (31.5)	*0.156*	47 (27.3)
Wartenberg’s sign	12 (18.8)	13 (12.0)	*0.266*	25 (14.5)
Ulnar nerve subluxation at the elbow	11 (17.2)	6 (5.6)	*0.018*	17 (9.9)
Claw hand	5 (7.8)	3 (2.8)	*0.150*	8 (4.7)

## Results

### Demographic Information

A total of 190 cases of ECuTR were performed in 166 patients between March 2003 and January 2010 by a single surgeon. Eighteen cases did not have positive preoperative electrical studies and were excluded from the study, leaving 172 cases in 148 patients. Mean age at time of surgery was 48 (range 24–82) years for women and 53 (range 22–90) years for men. There were 56 (38 %) women and 92 (62 %) men. Twenty-four patients underwent bilateral procedures, of the remaining 124, 56 (43 %) occurred on the dominant hand side. Bilateral surgery was performed in 8 (14 %) of the women and 16 (17 %) of the men with a median number of 35 days (range 0–579) between surgeries. Mean follow-up was 30 months (range 1–90). Kaplan-Meier analyses were used; therefore, minimum follow-up exclusions were not implemented because RTW time was expected to occur within days after surgery. Baseline characteristics of preoperative complaints and findings are shown in Table 1. Electrophysiologists classified the results of electrical studies as mild in 26 %, moderate in 39 %, and severe in 35 %. Of the 172 patients studied, 52 (30 %) were homemaker/retired/unemployed, 86 (50 %) were considered under manual labor, and 34 (20 %) under nonmanual labor. The mean tourniquet time was 10 min (range 3–23).

### Return to Full-Duty Work

**Fig. 1:**
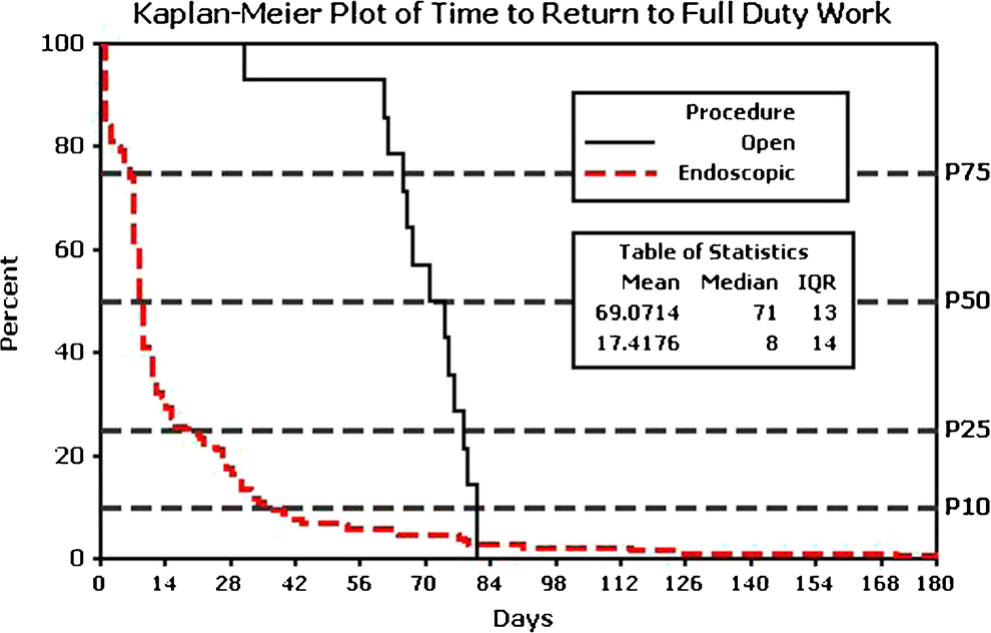
Kaplan-Maier survival curve showing return to full-duty work for ECuTR compared with ATUN. Note that half of the patients are back to full-duty work 8 days postoperatively following ECuTR versus 71 days following ATUN

Median time for RTW was significantly higher (*P* < 0.001) following ATUN (71 days) (interquartile range (IQR) = 13, 76–63 days) compared with ECuTR (8 days) (IQR = 10, 16–6 days; Fig. 1) with the variables of male sex (*P* = 0.006), manual labor (*P* < 0.001), and worker’s compensation status (*P* < 0.001) negatively affecting RTW following ECuTR.

### Satisfaction

Dellon’s was the only preoperative variable that significantly predicted postoperative satisfaction (*P* = 0.01; Table 2). As expected, ordinal logistic regression analysis showed that patients with resolution of their N/T and pain reported the highest postoperative satisfaction. The distribution of Bishop’s by Dellon’s class (Table 3) was significantly inversely correlated (*P* < 0.001).

**Table 2:** Postoperative satisfaction by preoperative Dellon’s class

Dellon’s class	Satisfaction^a^ rating of surgical outcome (*n* (%))				
	1	2	3	4	5
	(*Very dissatisfied*)				(*Very satisfied*)
1—mild	–	–	–	1 (5.0)	19 (95.0)
2—moderate	1 (1.5)	–	4 (5.9)	14 (20.6)	49 (72.1)
3—severe	3 (3.9)	–	6 (7.7)	21 (26.9)	48 (61.5)
Total	4 (2.4)	–	10 (6.0)	36 (21.7)	116 (69.9)

^a^Satisfaction scores were missing for six patients

**Table 3:** Postoperative Bishop’s rating by preoperative Dellon’s classes 1, 2, and 3

Dellon’s class	Modified Bishop’s rating system (*n* (%))			
	Poor	Fair	Good	Excellent
	*0 to 2*	*3 to 4*	*5 to 7*	*8 to 9*
1—mild	–	–	–	20 (100.0)
2—moderate	–	1 (1.4)	11 (15.7)	58 (82.9)
3—severe	–	6 (7.3)	17 (20.7)	59 (72.0)
Total	–	7 (4.1)	28 (16.3)	137 (79.7)

### Resolution Rates

**Fig. 2:**
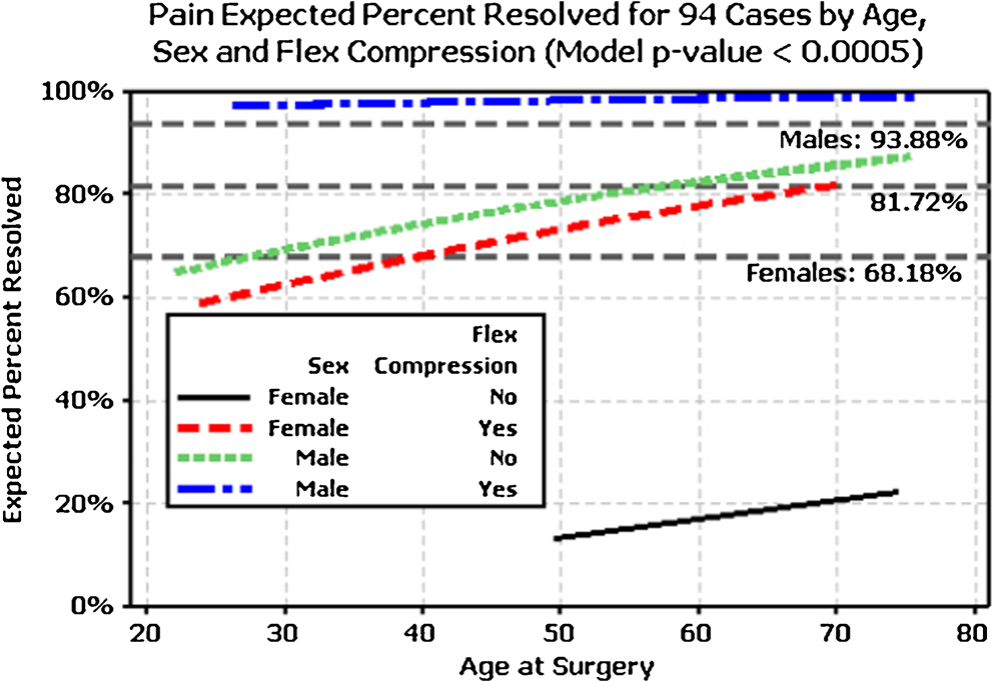
Expected percent of pain relief by age, sex, and flexion compression test (model *P* < 0.001). A higher percentage of pain resolution occurred with advancing age, male gender, and for patients with a positive flexion compression test

Younger patients with negative preoperative Wartenberg’s and positive ulnar nerve subluxation reported nearly 100 % resolution of N/T postoperatively (*P* = 0.006). Resolution rates of preoperative findings and complaints as well as significance by gender are shown in Table 4. Controlling for sex, the variation in the prevalence of pain at follow-up is explained by gender and is independent of pain at baseline. Pain was significantly negatively associated with static numbness (*P* = 0.001) and clawing (*P* = 0.004). Patients with a positive flexion compression test preoperatively had significantly better chances of resolution of pain (*P* < 0.001). The effect of gender by age and results of flexion compression test on pain resolution are shown in Fig. 2.

**Table 4:** Resolution rates of common preoperative findings and conditions by case

Preoperative condition	Women		Men		Overall	
	(*n* = 64 total cases)		(*n* = 108 total cases)		(*n* = 172 total cases)	
	*n*	*n* (%)	*n*	*n* (%)	*n*	*n* (%)
	Resolved		Resolved		Resolved	
Subjective complaints (ulnar nerve distribution)—*n* (%)						
Numbness/tingling	63	50 (79.4)	98	77 (78.6)	161	127 (78.9)
Pain^a^	45	30 (66.7)	49	46 (93.9)	94	76 (80.9)
Orthopedic exam (ulnar nerve distribution): positive findings—*n* (%)						
Finger abduction weakness	31	26 (83.9)	42	37 (88.1)	73	63 (86.3)
Two-point discrimination ≥6 mm	18	13 (72.2)	27	21 (77.8)	45	34 (75.6)
Hypothenar muscle atrophy	13	8 (61.5)	33	28 (84.9)	46	36 (78.3)
Wartenberg’s sign	8	5 (62.5)	12	8 (66.7)	20	13 (65.0)

Missing data if *n* is not equal to total number of cases

^a^Statistically significant difference in resolution of symptom between genders (*P* < 0.05)

### Ulnar Nerve Subluxation

The prevalence of preoperative ulnar nerve subluxation was significantly greater for women (17 %) than for men (6 %) (*P* = 0.02). Preoperative Dellon’s classification was not affected by the presence of subluxation (*P* = 0.26). Postoperative resolution rates of pain (*P* = 0.69), N/T (*P* = 0.53), and satisfaction (*P* = 0.37) were not affected by the presence of preoperative ulnar nerve subluxation.

### Complications and Failures

There were seven (4 %) postoperative complications. Four wound dehiscence were treated on an outpatient basis. One postoperative hematoma was resolved without intervention. Two cases of cellulitis responded to short courses of oral antibiotics. All complications were resolved without further sequela.

There were four (2 %) failures requiring revision surgery. Three were due to persistent symptoms, and one was due to recurrence at 4 months postoperatively. Three of the four failures had workman’s compensation claims. All underwent open revision surgery within 9 months of the original surgery. One patient who continued to complain of symptoms after revision surgery failed all of the validities on a functional capacity evaluation. None of the patients had complete resolution of their symptoms following revision surgery.

## Discussion

Patients frequently query their surgeon with questions about outcomes and “what is the chance” and “how soon can I” types of questions. This study was designed to quantify outcomes following ECuTR by determining the following: (1) RTW time and comparing these times with those of a cohort of patients undergoing ATUN, (2) satisfaction rates and factors affecting satisfaction, (3) resolution rates of common preoperative complaints and findings, and (4) effect of preoperative nerve subluxation on outcomes.

### Return to Full-Duty Work

While the RTW time was relatively short for the ECuTR group, our practice is to provide limited duty immediately and full duty in 1 week for manual occupations, thereby potentially artificially lengthening RTW time in some cases. Factors that the the authors would recommend to improve RTW time following surgery include enhanced patient self-efficacy practices through setting expectations for early RTW prior to surgery and striving to give limited duty permission (or full duty when possible) rather than off-work permission slips.

### Economic Benefits

Endoscopic methods have been criticized as unnecessarily expensive for a procedure that is easily performed open. However, it is important to consider both the direct and indirect costs of illness and the potential economic benefits associated with faster RTW times following ECuTR compared with ATUN. Indirect costs such as lost work productivity account for two thirds of the total cost of surgical treatment [40] and were recently estimated to be $260 billion annually [41]. Considering that the projected number of CuTS cases for the year 2016 is 73,673 [3], the results from our study that patients are back to work 63 days sooner following ECuTR compared with ATUN and that the estimated cost of disability in the USA is $94 per day [42], the possible annual savings for the USA in 2016 could be estimated to be $436 million (73,673 cases × 63 days × $94/day).

Additional savings could be estimated based on facility savings for decreased surgical times. The mean tourniquet time for our study was 10 min compared with a reported national time of 59 min for ATUN [41], a difference of 49 min. The estimated average cost of running an operating room is $20 per minute [43, 44]. Based on these numbers, there are potential additional savings of $72,199,540 (49 min × $20/min × 73,673 cases). Furthermore, the potential cost savings of anesthesia, billed as a base price plus $65 per 15-min time block at our surgery center, would be $14,366,235 based on the 2016 projections ($195 per case × 73,673 cases).

Additional costs for the endoscopic procedure, including equipment costs and surgeon-training time and expenses, are difficult to quantify. However, we estimated the total savings to society for conversion to the endoscopic method to be $522,565,775 for the year 2016 ($436 million of disability costs + $72,199,540 operating room time cost + $14,366,235 anesthesia costs—additional costs noted above). Furthermore, potential cost savings to payers based on mean hospital charges of $62 per minute of operating time [43] are $223,818,574 ($62/min × 49-min difference × 73,673 cases per year).

### Satisfaction

Preoperative Dellon’s classification was the best preoperative predictor of postoperative satisfaction. This is consistent with Watt’s and Bain’s who reported that the single factor that predicted reported satisfaction was preoperative function score [24]. Although their scale was different and follow-up shorter, our results of 92 % *mostly* or *very satisfied* compare favorably with their 79 % for endoscopic and 60 % for open in situ. Outcomes based on Bishop’s scores in our study were also correlated negatively with preoperative Dellon’s classification.

### Resolution Rates

Although the resolution rates of common preoperative complaints and findings for this study were fairly high, residual symptoms are apparently more common than one might think. In fact, some level of residual numbness has been reported in 53 % following ECuTR and 80 % for open in situ decompression [24]. As is consistent with other studies, resolution of preoperative pain was significantly less for women (67 %) compared with men (94 %) [45–47]. In our study, women with negative preoperative flexion compression tests had about a 20 % chance of resolving their pain compared with 98 % for men with positive preoperative flexion compression tests (Fig. 2). Advancing age was shown to be negatively associated with the presence of postoperative pain in our study as well as other studies [48].

### Ulnar Nerve Subluxation

Postoperative results based on satisfaction, pain, or N/T were not affected by preoperative ulnar nerve subluxation. These findings are similar to those of Bartels et al. [33] who found ulnar subluxation to have no effect on outcome following simple decompression. Our findings of 10 % ulnar nerve subluxation during elbow flexion are similar to those reported by Calfee et al. [37] who found 7 % in an evaluation of 400 asymptomatic volunteers. Although Calfee et al. [37] failed to demonstrate any significant difference in demographics based on ulnar nerve instability, it is unclear why we observed a higher incidence in women. Our findings suggest that ulnar nerve instability in the absence of ulnar neuritis does not mandate anterior transposition.

### Limitations

The results of this study are limited by the inclusion of a retrospectively reviewed nonrandomized and noncontrolled comparison of the ATUN study group. A single surgeon performed all surgical procedures, and RTW time may be influenced by RTW counseling, recommendations, and practice logistics that tend to limit RTW eligibility based on frequency of clinic visits. For example, a patient who had not returned to full duty and returns for their follow-up appointment a week later would delay the return to full duty by 1 week.

Another limitation of our study is that the assessment of ulnar nerve subluxation was based on physical examination and was not confirmed by intraoperative findings. Not only could our estimation of instability be incorrect based on preoperative findings, it is also unknown how many patients were potentially rendered unstable at the time of surgery. We are currently collecting data in a multicenter study that will allow assessment of outcomes following cubital tunnel release findings based on preoperative physical exam as well as intraoperative findings.
